# Elevated serum uric acid increases incident coronary artery calcification risk in Chinese adults undergoing health checkups

**DOI:** 10.3389/fcvm.2026.1861331

**Published:** 2026-07-15

**Authors:** You You, Qixiang Lei, Min Ni, Ke Ming, Yujun Long, Yu Lu

**Affiliations:** 1 Health Management Center, Zigong Fourth People's Hospital, Zigong, China; 2 Department of Scientific Research and Education, Zigong Fourth People's Hospital, Zigong, China; 3 Computer Center, Zigong Fourth People's Hospital, Zigong, China; 4 Department of Radiology, Zigong Fourth People's Hospital, Zigong, China

**Keywords:** cardiovascular risk, cohort study, coronary artery calcification, low-dose CT, serum uric acid

## Abstract

**Background:**

Coronary artery calcification is a key predictor of cardiovascular disease. However, the association between serum uric acid and incident coronary artery calcification remains controversial, and relevant evidence from Chinese populations is limited.

**Methods:**

We conducted a retrospective cohort study including 6,996 participants without coronary artery calcification at baseline, as detected by low-dose chest computed tomography. Multivariable Cox regression was performed to assess the association between serum uric acid and the risk of incident coronary artery calcification.

**Results:**

During a median follow-up of 6.0 years, 560 participants (8.0%) developed incident coronary artery calcification. Each standard deviation increase in serum uric acid was independently associated with a higher risk of incident coronary artery calcification [hazard ratio (HR): 1.21, 95% confidence interval (CI): 1.09–1.35, *P* < 0.001]. Participants with hyperuricemia also had a significantly higher risk compared with those with normal uric acid in the fully adjusted model (HR: 1.31, 95% CI: 1.08–1.58, *P* = 0.005). A linear dose–response association was observed between serum uric acid levels and the risk of incident coronary artery calcification.

**Conclusions:**

This large Chinese cohort study provides new evidence that elevated serum uric acid is independently associated with an increased risk of incident coronary artery calcification, supporting the potential value of serum uric acid management in the early prevention of coronary atherosclerosis.

## 1 Introduction

Coronary artery calcification (CAC) is a well-recognized imaging indicator of coronary atherosclerotic burden and has been firmly established as an independent and powerful predictor of cardiovascular events and all-cause mortality (1, 2). The Agatston score, based on electrocardiography-gated computed tomography (CT), remains the reference standard for quantitative CAC evaluation and provides critical information for cardiovascular risk stratification (3). Meanwhile, the widespread implementation of low-dose non-gated chest CT for lung cancer screening has created an unprecedented opportunity for the opportunistic detection of CAC without additional radiation exposure or healthcare costs (4, 5). The 2024 European Society of Cardiology (ESC) Guidelines for chronic coronary syndromes explicitly recommend incorporating existing CAC findings from prior chest CT scans to refine risk stratification and guide personalized management of modifiable cardiovascular risk factors (6). Accumulating evidence has confirmed that visual or semiquantitative CAC assessment using low-dose computed tomography (LDCT) correlates strongly with the Agatston score and effectively predicts adverse cardiovascular outcomes (7, 8). Thus, LDCT-based CAC evaluation offers incremental prognostic value and represents a practical, cost-effective strategy to improve cardiovascular risk assessment in individuals undergoing health evaluation or lung cancer screening.

Serum uric acid (sUA), the end product of purine metabolism, is closely associated with major cardiovascular risk factors including hypertension, insulin resistance, chronic kidney disease (CKD), and chronic inflammation (9, 10). Basic and epidemiological evidence suggests that hyperuricemia may promote the osteogenic phenotypic transformation of vascular smooth muscle cells and accelerate vascular calcification through mechanisms such as oxidative stress, inflammatory responses, and endothelial dysfunction (10, 11). However, controversy remains regarding whether uric acid is an independent risk factor for CAC. Several cohort studies and meta-analyses have reported a significant positive association (12, 13), whereas other studies have observed a weakened or null association after full adjustment for traditional cardiovascular risk factors (14–16). Furthermore, the interrelationships between uric acid, renal function, and metabolic diseases complicate causal inference (17).

Although the association between sUA and CAC has been widely investigated, results remain inconsistent, particularly in individuals undergoing LDCT for health evaluation or lung cancer screening. We therefore conducted the present study to explore the relationship between sUA and incident CAC, and to determine whether sUA serves as an independent risk factor for incident CAC in this population.

## 2 Materials and methods

### 2.1 Study design and participants

**Figure 1:**
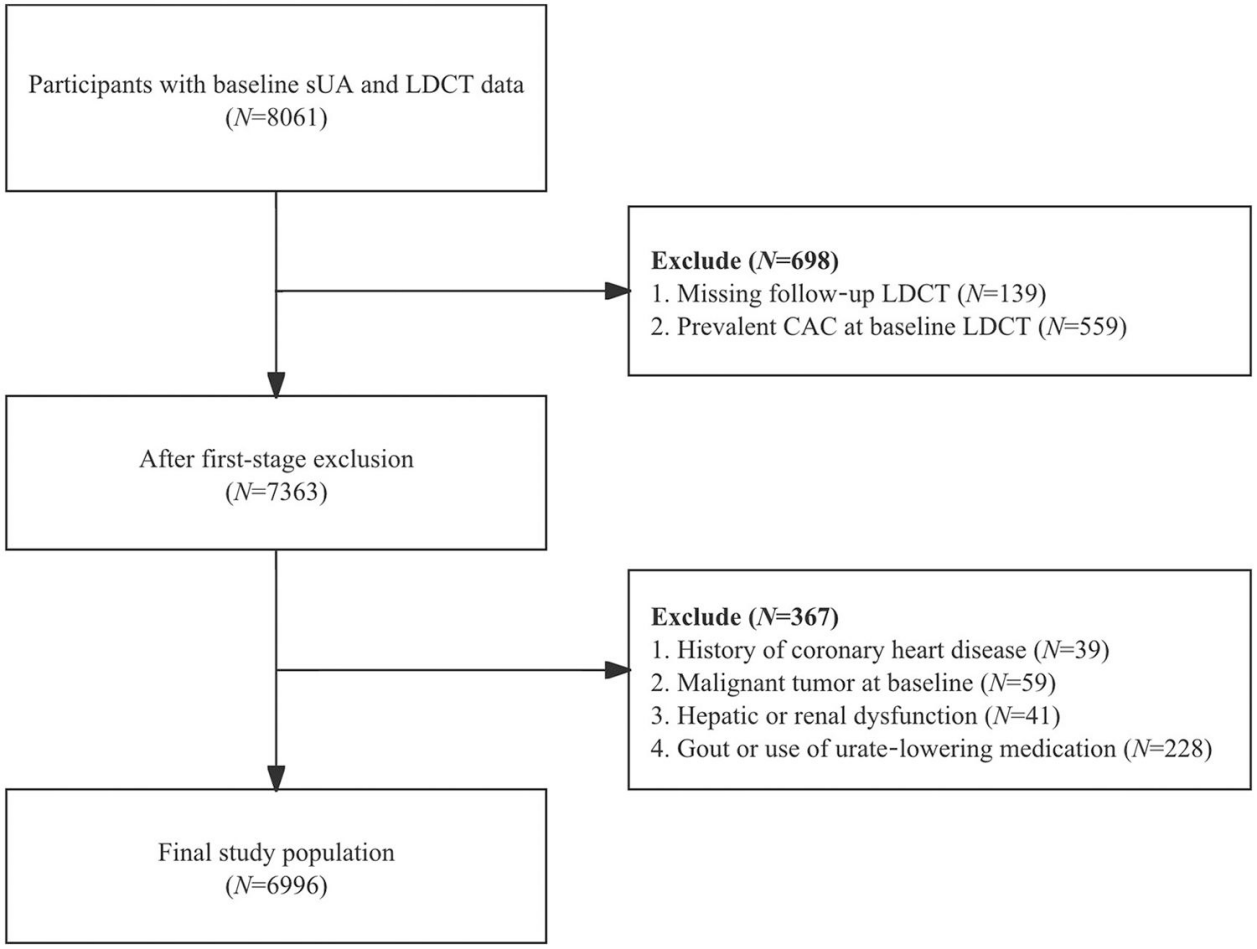
Flow diagram of selection of 8,061 participants initially assessed; 698 were excluded due to missing follow-up (*n* = 139) or prevalent CAC (*n* = 559). Among the remaining 7,363, a further 367 were excluded for coronary heart disease (*n* = 39), malignancy (*n* = 59), hepatic or renal dysfunction (*n* = 41), or gout/urate-lowering therapy (*n* = 228). The final cohort comprised 6,996 participants without baseline CAC.

This retrospective cohort study was conducted in accordance with the Strengthening the Reporting of Observational Studies in Epidemiology (STROBE) statement and the Declaration of Helsinki. Participants were enrolled from individuals undergoing routine health checkups at the Health Management Center of Zigong Fourth People's Hospital between 1 January and 31 December 2018. Follow-up extended from baseline until the first detection of incident CAC (event) or the date of the last available follow-up CT examination (censoring), whichever came first. The study follow-up period closed on 31 December 2024. The study protocol was approved by the Ethics Committee of Zigong Fourth People's Hospital (Approval Number: EC-2025-188). Written informed consent was waived due to the retrospective and anonymized study design. Inclusion criteria were (1) age ≥18 years at baseline and (2) availability of baseline serum uric acid and low-dose chest computed tomography data. Participants were excluded if they (1) had missing follow-up imaging data, (2) had prevalent coronary artery calcification at baseline, (3) had a history of coronary heart disease, (4) had a malignant tumor at baseline, (5) had hepatic or renal dysfunction, or (6) had gout or used urate-lowering medications. A total of 8,061 participants were initially assessed, and 6,996 participants were finally included after applying all exclusion criteria. The participant flow diagram is shown in Figure 1.

### 2.2 Data collection

Clinical data for all participants were extracted from the center's electronic database, including demographic characteristics, lifestyle factors, anthropometric measurements, medical history, laboratory test results, and imaging data. The specific variables collected were as follows: (1) demographic characteristics: age, sex; (2) lifestyle factors: smoking status, alcohol consumption status; (3) anthropometric measurements: height, weight, systolic blood pressure (SBP), diastolic blood pressure (DBP); (4) medical history: history of diabetes mellitus and hypertension; and (5) laboratory test results: sUA, lipid profile [low-density lipoprotein cholesterol (LDL-C), high-density lipoprotein cholesterol (HDL-C), total cholesterol (TC), triglycerides (TG)], fasting plasma glucose (FPG), serum creatinine (Scr), and alanine transaminase (ALT).

### 2.3 Exposure and outcome definitions

The exposure variable was baseline sUA (μmol/L), analyzed both as a continuous variable [per 1-standard deviation (SD) increment] and as a categorical variable based on sex-specific thresholds recommended by the 2024 Chinese guidelines for hyperuricemia: ≥ 420 μmol/L for men and ≥ 360 μmol/L for women (18). The primary endpoint was incident CAC. All participants underwent CAC assessment using LDCT as part of their annual routine health checkup, with a median interscan interval of 1.3 years. Scans were acquired using one of two Siemens multidetector row CT scanners (Germany) during a single breath-hold at end-inspiration. Scanning parameters included tube voltage 150 kV with tin filtration (Sn150) and automatic tube current modulation. CAC was visually assessed as present or absent by two chest radiologists blinded to all clinical data and sUA levels. Each reviewer had at least 5 years of experience in chest imaging; the second radiologist had access to the initial report. The double-reading consistency between the two readers was 99.66%. Visual assessment of coronary calcification is a validated and widely used method in LDCT studies (5, 7). CAC was defined as any visually detected hyperdense calcified lesion within the four major coronary arteries and their branches: left main, left anterior descending, left circumflex, and right coronary artery. Calcifications in the aortic wall, aortic valve, mitral annulus, coronary sinus, pulmonary vessels, or lung parenchyma were explicitly excluded.

### 2.4 Variable definitions

Hypertension was defined as a previous diagnosis by a physician, current use of antihypertensive medications, systolic blood pressure ≥140 mmHg, or diastolic blood pressure ≥90 mmHg. Diabetes mellitus was defined as a previous diagnosis by a physician, current use of antidiabetic agents, or FPG ≥ 7.0 mmol/L. HbA1c was not routinely measured in the health checkup panel during the study period and therefore was not used in the definition. Smoking status was categorized as current, former, or never smoker. Alcohol consumption was classified as current drinker or non-drinker. Body mass index (BMI) was calculated as weight (kg) divided by height squared (m^2^). Estimated glomerular filtration rate (eGFR) was calculated using the Chronic Kidney Disease Epidemiology Collaboration (CKD-EPI) Creatinine Equation (19). Renal dysfunction was defined as eGFR <60 mL·min⁻^1^·1.73 m⁻^2^. Hepatic dysfunction was defined as ALT >5 times the upper limit of normal (20).

### 2.5 Statistical analysis

Normality of data distribution was assessed using the Kolmogorov–Smirnov test. Normally distributed data were presented as mean ± SD, while non-normally distributed data were expressed as median (interquartile range, IQR). Between-group comparisons were performed using the *t*-test, Mann–Whitney *U* test, or *χ*^2^ test, as appropriate. The maximum number of missing values for any variable was 1,778 (25.4%), with detailed information on missing data presented in Supplementary Table 1. Missing data were handled using multiple imputation by chained equations (MICE, *m* = 10), and the estimates were pooled across the imputed datasets. The association between sUA and incident CAC was evaluated using Cox proportional hazards (PH) models. Prior to model fitting, the PH assumption was tested using Schoenfeld residuals (Supplementary Table 2 and Supplementary Figure 1) and confirmed to be valid. Three hierarchical models were constructed: Model 1 (unadjusted); Model 2 (adjusted for age, sex, hypertension, and diabetes mellitus); and Model 3 (further adjusted for smoking status, alcohol consumption, BMI, SBP, FPG, TG, TC, LDL-C, and eGFR). Covariates were selected based on clinical relevance and prior literature. Multicollinearity among these covariates was evaluated using generalized variance inflation factors (GVIF), standardized as GVIF^[1/(2·df)], with values >2 indicating meaningful multicollinearity. Detailed results of multicollinearity testing—as presented in Supplementary Table 3—confirmed that no meaningful multicollinearity existed among the included covariates. Dose–response relationships between sUA and incident CAC were assessed using restricted cubic splines (RCS), and subgroup analyses were conducted to examine effect heterogeneity. Sensitivity analyses included (1) propensity score matching (PSM) (1:1 nearest-neighbor matching with a caliper of 0.2 SD of the propensity score); (2) complete case analysis (excluding participants with missing data); and (3) time-varying Cox regression analysis (sUA updated at each follow-up visit; intervals with missing sUA excluded without imputation). All statistical analyses were performed using R software (version 4.5.2) and the Free Statistics platform. A two-sided *P*-value < 0.05 was considered statistically significant.

## 3 Results

### 3.1 Baseline characteristics of study participants

Baseline characteristics of the 6,996 participants according to sUA status are presented in Table 1. The mean age was 47.1 ± 10.3 years, and 62.5% were men. Compared with the normal sUA group (*n* = 5,173), the hyperuricemia group (*n* = 1,823) had a higher proportion of men, current smokers, drinkers; and greater hypertension prevalence; higher BMI, SBP, DBP, TC, TG, Scr, LDL-C, and ALT; and lower HDL-C and eGFR (all *P* < 0.001). FPG was also higher in the hyperuricemia group (*P* = 0.021). There were no significant between-group differences in age or diabetes prevalence (all *P* > 0.05).

**Table 1:** Baseline characteristics of the study population.

Variables	Total (*n* = 6,996)	Normal sUA (*n* = 5,173)	Hyperuricemia (*n* = 1,823)	*P*
Age (years)	47.1 ± 10.3	47.2 ± 10.2	47.0 ± 10.5	0.426
Male sex, *n* (%)	4,374 (62.5)	2,831 (54.7)	1,543 (84.6)	<0.001
Incident CAC, *n* (%)	560 (8.0)	360 (7.0)	200 (11.0)	<0.001
Smoking, *n* (%)				<0.001
Never	4,662 (66.6)	3,636 (70.3)	1,026 (56.3)	
Former	208 (3.0)	122 (2.4)	86 (4.7)	
Current	2,126 (30.4)	1,415 (27.4)	711 (39.0)	
Drinking, *n* (%)	2,874 (41.1)	1,789 (34.6)	1,085 (59.5)	<0.001
Hypertension, *n* (%)	1,181 (16.9)	715 (13.8)	466 (25.6)	<0.001
Diabetes, *n* (%)	394 (5.6)	286 (5.5)	108 (5.9)	0.529
BMI (kg/m^2^)	23.5 ± 3.1	23.0 ± 2.9	25.1 ± 3.0	<0.001
SBP (mmHg)	117.7 ± 16.9	115.9 ± 16.6	122.8 ± 16.7	<0.001
DBP (mmHg)	72.5 ± 11.5	71.0 ± 11.2	76.6 ± 11.5	<0.001
Scr (μmol/L)	64.2 ± 14.0	61.8 ± 13.4	71.2 ± 13.0	<0.001
eGFR (mL/min/1.73m^2^)	109.8 ± 10.5	110.7 ± 9.9	107.1 ± 11.7	<0.001
FPG (mmol/L)	5.1 ± 1.3	5.1 ± 1.3	5.1 ± 1.1	0.021
TC (mmol/L)	4.9 ± 0.9	4.8 ± 0.9	5.0 ± 0.9	<0.001
TG (mmol/L)	1.4 (0.9, 2.1)	1.2 (0.9, 1.8)	2.0 (1.4, 3.0)	<0.001
HDL-C (mmol/L)	1.3 ± 0.4	1.3 ± 0.4	1.1 ± 0.3	<0.001
LDL-C (mmol/L)	2.5 ± 0.7	2.5 ± 0.6	2.6 ± 0.7	<0.001
ALT (U/L)	24.0 (17.0, 36.0)	22.0 (16.0, 32.0)	32.0 (22.0, 46.0)	< 0.001

Data are presented as mean ± SD for normally distributed continuous variables, median (IQR) for non-normally distributed continuous variables, and *n* (%) for categorical variables. Results are based on the imputed dataset.

CAC, coronary artery calcification; BMI, body mass index; SBP, systolic blood pressure; DBP, diastolic blood pressure; Scr, serum creatinine; eGFR, estimated glomerular filtration rate; FPG, fasting plasma glucose; TC, total cholesterol; TG, triglyceride; HDL-C, high-density lipoprotein cholesterol; LDL-C, low-density lipoprotein cholesterol; ALT, alanine transaminase.

### 3.2 Association of sUA with incident CAC

Over a median follow-up of 6.0 years, 560 participants (8.0%) developed incident CAC. The associations of sUA with incident CAC are presented in Table 2. When analyzed as a continuous variable (per 1-SD increment), elevated sUA was significantly associated with a higher risk of incident CAC in the unadjusted model [Model 1: hazard ratio (HR) = 1.39, 95% confidence interval (CI): 1.29–1.50, *P* < 0.001], and this association remained statistically significant in the fully adjusted model (Model 3: HR = 1.21, 95% CI: 1.09–1.35, *P* < 0.001). For categorical analysis, compared with the normal sUA group, participants with hyperuricemia exhibited a significantly higher risk of incident CAC across all models, with a fully adjusted HR of 1.31 (95% CI: 1.08–1.58, *P* = 0.005).

**Table 2:** Multivariable Cox regression analysis of sUA and incident CAC.

Variable	Total, *n*	Event, *n* (%)	Model 1		Model 2		Model 3	
			HR (95% CI)	*P*	HR (95% CI)	*P*	HR (95% CI)	*P*
sUA (Per SD)	6,996	560 (8.0)	1.39 (1.29–1.50)	<0.001	1.21 (1.10–1.33)	<0.001	1.21 (1.09–1.35)	<0.001
Groups								
Normal sUA	5,173	360 (7.0)	1.00 (Ref)		1.00 (Ref)		1.00 (Ref)	
Hyperuricemia	1,823	200 (11.0)	1.62 (1.36–1.93)	<0.001	1.34 (1.12–1.60)	0.001	1.31 (1.08–1.58)	0.005

HR, hazard ratio; CI, confidence interval. Model 1: Unadjusted; Model 2: Adjusted for sex, age, history of hypertension, and diabetes; Model 3: Further adjusted for smoking, drinking, BMI, SBP, FPG, TG, TC, LDL-C, and eGFR.

### 3.3 Dose–response relationship

**Figure 2:**
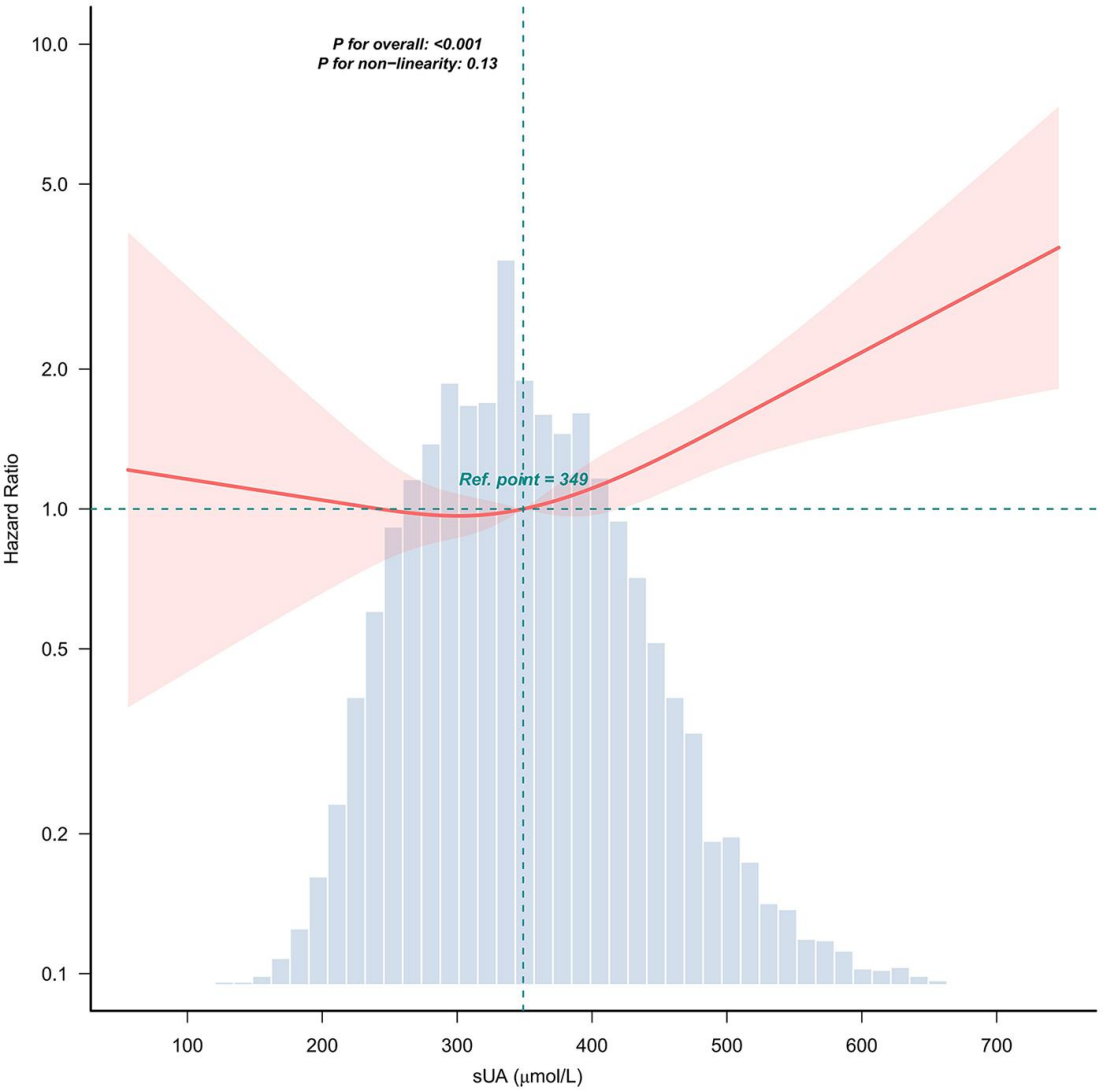
Linear association between sUA and incident CAC. The dose–response association was assessed using RCS with four knots placed at the 5th, 35th, 65th, and 95th percentiles of baseline sUA (reference: median, 349 μmol/L). The solid line represents the hazard ratio, and the shaded area represents the 95% CI. The model was adjusted for age, sex, hypertension, diabetes, smoking, drinking, BMI, SBP, FPG, TG, TC, LDL-C, and eGFR (Model 3). The overall association was statistically significant (*P* for overall < 0.001), while the test for non-linearity was not significant (*P* for non-linearity = 0.13), indicating an approximately linear dose–response relationship. The histogram along the *x*-axis shows the distribution of baseline sUA values.

The dose–response association between sUA and incident CAC was assessed using restricted cubic splines in the fully adjusted model (Figure 2). The overall association was statistically significant (*P* for overall < 0.001), while the non-linearity test yielded a non-significant result (*P* for non-linearity = 0.13).

### 3.4 Subgroup analyses

**Figure 3:**
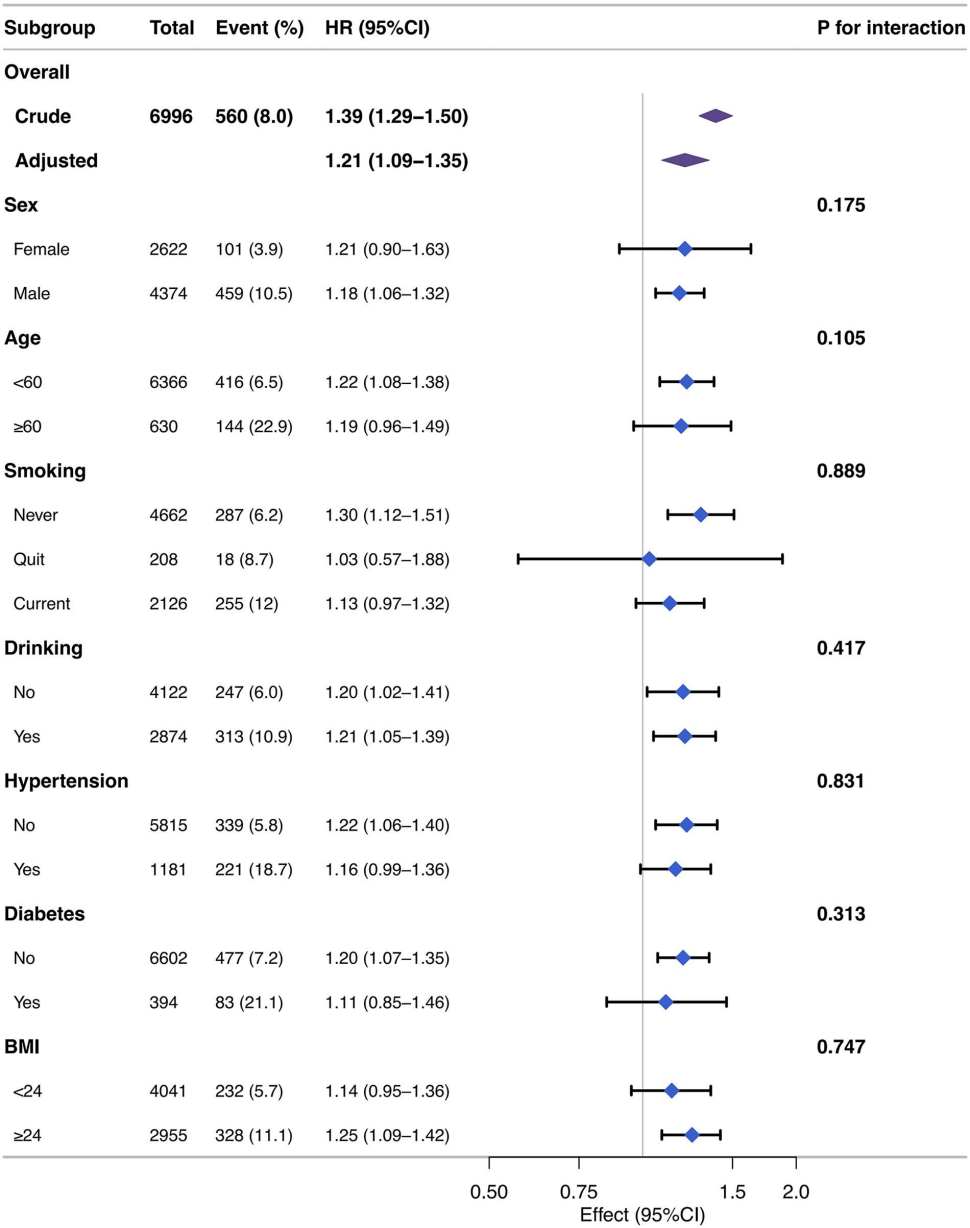
Subgroup analyses of the association between sUA and incident CAC. Hazard ratios (diamonds) and 95% confidence intervals (horizontal lines) were estimated using stratified Cox regression models within each subgroup, with sUA modeled as a continuous variable (per 1-SD increase). BMI was stratified at 24 kg/m^2^ and age at 60 years according to conventional clinical thresholds. All models were adjusted for the same covariates as Model 3 (age, sex, hypertension, diabetes, smoking, drinking, BMI, SBP, FPG, TG, TC, LDL-C, and eGFR), except for the stratification variable itself. The dashed vertical line at HR = 1.0 indicates no association. *P* values for interaction were used to evaluate effect modification; no significant interactions were observed (all *P* for interaction > 0.05).

Subgroup analyses were conducted across strata of sex, age (<60 vs. ≥60 years), smoking status, alcohol drinking, hypertension, diabetes, and BMI (<24 vs. ≥24 kg/m^2^) (Figure 3). No significant effect modification was identified between serum uric acid and any stratified factor (all *P* for interaction >0.05).

### 3.5 Sensitivity analyses

To validate the robustness of our primary findings, three sensitivity analyses were conducted. First, PSM was performed to balance major confounding covariates between sUA groups. After PSM, all standardized mean differences were less than 0.1, indicating satisfactory balance (Supplementary Table 4 and Supplementary Figure 2). The results of PSM-adjusted Cox regression are presented in Table 3. In the PSM sample, the fully adjusted model confirmed that per 1-SD elevation in sUA was significantly associated with a higher risk of incident CAC (HR = 1.30, 95% CI: 1.14–1.47, *P* < 0.001), and hyperuricemia remained an independent risk factor (HR = 1.34, 95% CI: 1.08–1.66, *P* = 0.009). Second, a complete case analysis was performed among participants with full data, yielding results consistent with the main analyses (Supplementary Table 5). Third, a time-varying Cox regression analysis was performed, and the results remained consistent with the main analyses (Supplementary Table 6).

**Table 3:** Multivariable Cox regression analysis of sUA and incident CAC after propensity score matching.

Variable	Total, *n*	Event, *n* (%)	Model 1		Model 2		Model 3	
			HR (95% CI)	*P*	HR (95% CI)	*P*	HR (95% CI)	*P*
sUA (Per SD)	3,258	338 (10.4)	1.29 (1.15–1.45)	<0.001	1.28 (1.14–1.45)	<0.001	1.30 (1.14–1.47)	<0.001
Group								
Normal sUA	1,629	154 (9.5)	1.00 (Ref)		1.00 (Ref)		1.00 (Ref)	
Hyperuricemia	1,629	184 (11.3)	1.23 (0.99–1.98)	0.061	1.30 (1.05–1.61)	0.016	1.34 (1.08–1.66)	0.009

HR, hazard ratio; CI, confidence interval. Model 1: Unadjusted; Model 2: Adjusted for sex, age, history of hypertension, and diabetes; Model 3: Further adjusted for smoking, drinking, BMI, SBP, FPG, TG, TC, LDL-C, and eGFR.

## 4 Discussion

This retrospective cohort study demonstrated that elevated sUA was independently associated with an increased risk of incident CAC among asymptomatic individuals undergoing LDCT for health evaluation or lung cancer screening. Both continuous sUA (per 1-SD increment) and hyperuricemia were significant risk predictors of incident CAC after full adjustment for confounders. Restricted cubic spline analysis confirmed an approximately linear dose–response relationship, and the results remained consistent in sensitivity analyses including PSM, complete case analysis, and time-varying Cox regression.

Our results are consistent with previous epidemiological studies that have identified a positive association between sUA and the presence or progression of CAC (21, 22). Similar linear or graded relationships have also been observed, supporting our finding that CAC risk rises continuously with increasing sUA levels. Although some studies failed to identify an independent association (14, 23), such discrepancies may be attributed to differences in population characteristics (e.g., CKD stage), study design, CAC assessment methods, and covariate adjustment sets. The present study provides novel evidence from a large Chinese population, which has been seldom reported in earlier research. Of note, the magnitude of the positive association between sUA and CAC was numerically larger in men than in women in the present study. This sex-specific difference is consistent with prior studies reporting stronger effects of sUA on cardiovascular outcomes in men, which may be partly explained by the uricosuric effects of estrogen and lower baseline sUA levels in women (15, 16). From a clinical perspective, sUA is a low-cost, widely available biomarker that can be readily incorporated into routine cardiovascular risk assessment. In populations undergoing LDCT for lung cancer screening or general health checkups, combining sUA measurement with opportunistic CAC detection may enable improved early risk stratification without additional radiation or cost (24). The linear dose–response pattern further supports the use of sUA as a continuous marker rather than relying solely on fixed hyperuricemia cutoffs.

Biologically, elevated sUA may promote coronary calcification through mechanisms including endothelial dysfunction, oxidative stress, inflammatory activation, and osteogenic phenotypic transformation of vascular smooth muscle cells (25–27). These pathways actively promote osteogenic transformation and calcium phosphate deposition in the coronary intima, thereby driving the development and progression of CAC. This mechanistic framework aligns with the independent association between sUA and incident CAC observed in the present study.

Several limitations should be acknowledged. First, this was a single-center retrospective study, which may limit generalizability, though our large sample and standardized procedures helped ensure data reliability. Second, CAC was assessed visually rather than by a formal scoring system, and the two radiologists were not fully independent. These factors may reduce quantitative precision, but our findings remain consistent with clinical practice. Third, unmeasured factors such as diet or physical activity may remain as residual confounders, although we adjusted for major cardiovascular risk factors in the analysis. Finally, as an observational study, we cannot establish causality, but our results align with well-established biological mechanisms linking metabolic factors to coronary calcification.

## 5 Conclusion

This study confirms that higher sUA is independently associated with an increased risk of incident CAC. A linear, dose–response relationship exists between uric acid levels and incident coronary calcification. These findings support sUA as a practical and readily available biomarker for early cardiovascular risk stratification in asymptomatic individuals undergoing low-dose CT screening.

## Data Availability

The raw data supporting the conclusions of this article will be made available by the authors, without undue reservation.
