# Supplementary material for: Elevated serum uric acid increases incident coronary artery calcification risk in Chinese adults undergoing health checkups
Source: Front Cardiovasc Med. 2026 Jul 15;13:1861331. doi: 10.3389/fcvm.2026.1861331 (PMC13416316; doi:10.3389/fcvm.2026.1861331)
Supplement: Supplementary file 1 [file Datasheet1.docx]

Supplementary Material

# Supplementary Figures

Supplementary Figure 1. Schoenfeld residual plots for testing the proportional hazards assumption of the Cox model. Beta coefficients for all variables remained stable over time, with all *P* > 0.05, indicating no violation of the proportional hazards assumption.





Supplementary Figure 2. Standardized mean differences (SMDs) of covariates before and after propensity score matching. SMDs were calculated to assess the balance between normal uric acid and hyperuricemia groups. A value of SMD < 0.1 was considered indicative of satisfactory balance. All covariates were well balanced after matching.


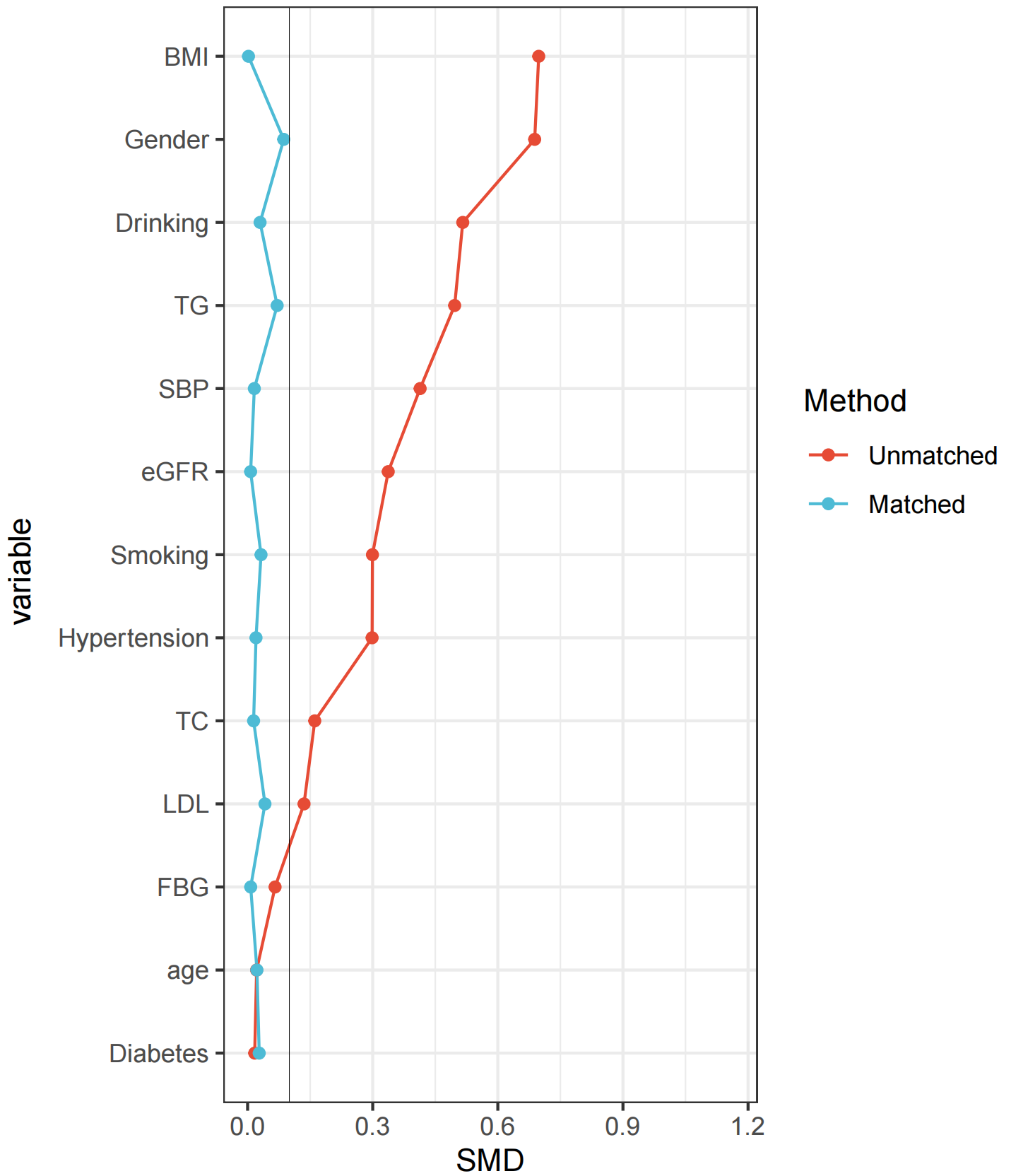


# Supplementary Tables

Supplementary Table 1. Missing data distribution of covariates.

| Variable | Miss. freq | Miss. Percentage (%) |
| --- | --- | --- |
| Drinking | 1778 | 25.4 |
| Smoking | 1777 | 25.4 |
| BMI | 885 | 12.7 |
| SBP | 799 | 11.4 |
| DBP | 799 | 11.4 |
| Hypertension | 643 | 9.2 |
| Diabetes | 624 | 8.9 |
| HDL | 30 | 0.4 |
| LDL | 30 | 0.4 |
| TC | 30 | 0.4 |
| TG | 30 | 0.4 |
| FPG | 28 | 0.4 |
| ALT | 14 | 0.2 |

BMI, body mass index; SBP, systolic blood pressure; DBP, diastolic blood pressure; HDL, high-density lipoprotein cholesterol; LDL, low-density lipoprotein cholesterol; TC, total cholesterol; TG, triglycerides; FPG, fasting plasma glucose; ALT, alanine aminotransferase.

Supplementary Table 2. Results of Schoenfeld residual test for proportional hazards assumption.

| Variable | chisq | df | *P* |
| --- | --- | --- | --- |
| sUA | 0.506 | 1 | 0.477 |
| Gender | 0.826 | 1 | 0.363 |
| Age | 0.414 | 1 | 0.52 |
| Hypertension | 0.010 | 1 | 0.922 |
| Diabetes | 0.000 | 1 | 0.991 |
| Smoking | 0.003 | 1 | 0.958 |
| Drinking | 0.158 | 1 | 0.691 |
| BMI | 3.295 | 1 | 0.069 |
| SBP | 0.173 | 1 | 0.677 |
| FPG | 0.182 | 1 | 0.67 |
| TG | 0.028 | 1 | 0.867 |
| LDL | 0.332 | 1 | 0.564 |
| TC | 0.137 | 1 | 0.711 |
| eGFR | 1.090 | 1 | 0.296 |
| Global | 10.712 | 15 | 0.773 |

sUA, serum uric acid; BMI, body mass index; SBP, systolic blood pressure; FPG, fasting plasma glucose; TC, total cholesterol; TG, triglycerides; LDL, low-density lipoprotein cholesterol; eGFR, estimated glomerular filtration rate.

Supplementary Table 3. GVIF values for assessing multicollinearity among covariates.

| Variable | GVIF | DF | GVIF^(1/(2*DF)) | Collinearity |
| --- | --- | --- | --- | --- |
| Gender | 1.8 | 1.0 | 1.4 | No |
| Age | 2.3 | 1.0 | 1.5 | No |
| Smoking | 1.4 | 2.0 | 1.1 | No |
| Drinking | 1.5 | 1.0 | 1.2 | No |
| Hypertension | 2.1 | 1.0 | 1.4 | No |
| Diabetes | 2.1 | 1.0 | 1.5 | No |
| BMI | 1.3 | 1.0 | 1.1 | No |
| SBP | 2.2 | 1.0 | 1.5 | No |
| FPG | 2.3 | 1.0 | 1.5 | No |
| TC | 4.1 | 1.0 | 2.0 | No |
| TG | 2.8 | 1.0 | 1.7 | No |
| LDL | 3.8 | 1.0 | 1.9 | No |
| eGFR | 2.2 | 1.0 | 1.5 | No |

BMI, body mass index; SBP, systolic blood pressure; FPG, fasting plasma glucose; TC, total cholesterol; TG, triglycerides; LDL, low-density lipoprotein cholesterol; eGFR, estimated glomerular filtration rate.

Supplementary Table 4. Baseline characteristics of the study population after propensity score matching.

| Variables | Total  (n = 3258) | Normal sUA  (n = 1629) | Hyperuricemia  (n = 1629) | *P* | SMD |
| --- | --- | --- | --- | --- | --- |
| Age(years) | 47.5 ± 10.2 | 47.6 ± 9.8 | 47.4 ± 10.5 | 0.524 | 0.022 |
| Gender, n (%) |  |  |  | 0.014 | 0.087 |
| Female | 505 (15.5) | 227 (13.9) | 278 (17.1) |  |  |
| Male | 2753 (84.5) | 1402 (86.1) | 1351 (82.9) |  |  |
| Incident CAC, n (%) | 338 (10.4) | 154 (9.5) | 184 (11.3) | 0.085 |  |
| Smoking, n (%) |  |  |  | 0.657 | 0.032 |
| Never | 1806 (55.4) | 890 (54.6) | 916 (56.2) |  |  |
| Former | 147 (4.5) | 75 (4.6) | 72 (4.4) |  |  |
| Current | 1305 (40.1) | 664 (40.8) | 641 (39.3) |  |  |
| Drinking, n (%) |  |  |  | 0.394 | 0.030 |
| No | 1360 (41.7) | 668 (41.0) | 692 (42.5) |  |  |
| Yes | 1898 (58.3) | 961 (59.0) | 937 (57.5) |  |  |
| Hypertension, n (%) | 782 (24.0) | 398 (24.4) | 384 (23.6) | 0.566 | 0.020 |
| Diabetes, n (%) | 205 (6.3) | 108 (6.6) | 97 (6.0) | 0.427 | 0.028 |
| BMI (kg/m²) | 24.8 ± 2.9 | 24.8 ± 2.9 | 24.8 ± 2.9 | 0.953 | 0.002 |
| SBP (mmHg) | 122.0 ± 16.8 | 122.1 ± 17.1 | 121.9 ± 16.5 | 0.646 | 0.016 |
| eGFR (mL/min/1.73m²) | 107.6 ± 10.9 | 107.6 ± 10.6 | 107.7 ± 11.2 | 0.836 | 0.007 |
| FPG (mmol/L) | 5.1 ± 1.2 | 5.1 ± 1.3 | 5.1 ± 1.0 | 0.833 | 0.007 |
| TC (mmol/L) | 4.9 ± 0.9 | 4.9 ± 0.9 | 4.9 ± 0.9 | 0.682 | 0.014 |
| TG (mmol/L) | 1.8 (1.2, 2.7) | 1.7 (1.1, 2.5) | 1.9 (1.3, 2.8) | < 0.001 | 0.071 |
| LDL-C (mmol/L) | 2.6 ± 0.7 | 2.6 ± 0.7 | 2.6 ± 0.7 | 0.235 | 0.042 |

sUA, serum uric acid; SMD, standardized mean difference; CAC, coronary artery calcification; BMI, body mass index; SBP, systolic blood pressure; eGFR, estimated glomerular filtration rate; FPG, fasting plasma glucose; TC, total cholesterol; TG, triglycerides; LDL-C, low-density lipoprotein cholesterol.

Supplementary Table 5. Association of sUA with incident CAC in complete-case analysis.

| Variable | Total, n | Event, n (%) | Model 1 | |  | Model 2 | |  | Model 3 | |
| --- | --- | --- | --- | --- | --- | --- | --- | --- | --- | --- |
|  |  |  | HR (95%CI) | *P* |  | HR (95%CI) | *P* |  | HR (95%CI) | *P* |
| Continuous |  |  |  |  |  |  |  |  |  |  |
| sUA (Per SD) | 4991 | 383 (7.7) | 1.42 (1.30-1.57) | <0.001 |  | 1.22 (1.09-1.37) | <0.001 |  | 1.24 (1.10-1.41) | 0.001 |
| Group |  |  |  |  |  |  |  |  |  |  |
| Normal sUA | 3686 | 241 (6.5) | 1.00 (Ref) |  |  | 1.00 (Ref) |  |  | 1.00 (Ref) |  |
| Hyperuricemia | 1305 | 142 (10.9) | 1.70 (1.38-2.09) | <0.001 |  | 1.41 (1.14-1.74) | 0.002 |  | 1.40 (1.12-1.75) | 0.003 |

HR, hazard ratio; CI, confidence interval. Model 1: Unadjusted; Model 2: Adjusted for sex, age, history of hypertension, and diabetes; Model 3: Further adjusted for smoking, drinking, BMI, SBP, FPG, TG, TC, LDL‑C, and eGFR

Supplementary Table 6. Association of time-varying sUA with incident CAC

| Variable | Model 1 | |  | Model 2 | |  | Model 3 | |
| --- | --- | --- | --- | --- | --- | --- | --- | --- |
|  | HR (95%CI) | *P* |  | HR (95%CI) | *P* |  | HR (95%CI) | *P* |
| Continuous |  |  |  |  |  |  |  |  |
| sUA (Per SD) | 1.34 (1.24-1.45) | <0.001 |  | 1.18 (1.06-1.30) | 0.002 |  | 1.14 (1.03-1.27) | 0.012 |
| Group |  |  |  |  |  |  |  |  |
| Normal sUA | 1.00 (Ref) |  |  | 1.00 (Ref) |  |  | 1.00 (Ref) |  |
| Hyperuricemia | 1.54 (1.29-1.83) | <0.001 |  | 1.34 (1.12-1.61) | 0.001 |  | 1.27 (1.05-1.53) | 0.012 |

HR, hazard ratio; CI, confidence interval. Model 1: Unadjusted; Model 2: Adjusted for sex, age, history of hypertension, and diabetes; Model 3: Further adjusted for smoking, drinking, BMI, SBP, FPG, TG, TC, LDL‑C, and eGFR. All covariates in Models 2 and 3 were assessed at baseline and treated as time-fixed variables. sUA was updated at each follow-up visit as a time-varying exposure; intervals with missing sUA were excluded (21,824 intervals from 6,996 participants with 520 incident CAC events).
